# Self‐Care for Sexual and Reproductive Health: Results From the French 2023 National Survey on Sexuality and Sexual Health

**DOI:** 10.1111/psrh.70074

**Published:** 2026-09-09

**Authors:** Caroline Moreau, Armelle Andro

**Affiliations:** ^1^ Soins Primaires et Prevention, CESP Center for Research in Epidemiology and Population Health, INSERM Villejuif France; ^2^ Department of Population, Family and Reproductive Health Johns Hopkins University Bloomberg School of Public Health Baltimore Maryland USA; ^3^ Paris 1 Pantheon‐Sorbonne University Paris France

## Abstract

**Introduction:**

Digital platforms have accelerated sexual and reproductive health (SRH) self‐care practices, but evidence is limited regarding the demand and usage of these resources and their potential to reduce SRH inequities.

**Methods:**

We draw on the CSF‐2023 study, a national cross‐sectional survey on sexuality in France. Our sample included 6305 sexually active individuals aged 18–49 who reported on SRH digital health care practices. We conducted logistic regressions to identify factors influencing the acceptability, information‐seeking, and purchasing of services online across SRH domains (oral contraception, medication abortion pills, sexually transmitted infection/HIV treatment, and sexual enhancement).

**Results:**

Men demonstrated higher acceptance of SRH online self‐care than women (59% vs. 53%), although overall acceptability was low (ranging from 10% for medication abortion pills to 23% for HIV Prep). Women sought more information about any topic (75% vs. 70%) and contraception and abortion specifically, while men focused more on sexual enhancement. Younger, more educated respondents and those with multiple partners or who met partners online sought a wider range of information. Acceptance was greater among men who had sex with men and respondents who reported sexual violence or unintended pregnancies, whereas rural residents were less accepting and sought less information. Recent medical care correlated with lower acceptance but did not affect information‐seeking.

**Conclusion:**

This study highlights a limited interest in digital prescription‐free SRH services in France, despite increasing reliance on digital platforms for SRH information. Targeted programmatic and policy investments are needed to bridge the digital divide, improve service quality, and explore alternative in‐person channels for SRH self‐care needs.

## RÉSUMÉ

**Introduction:**

Les plateformes numériques ont accéléré l'adoption des pratiques d'autos‐soins en santé sexuelle et reproductive (SSR) mais les connaissances sur la demande et l'utilisation de ces ressources, ainsi que sur leur potentiel à atténuer les inégalités demeurent limitées.

**Méthodes:**

Cette étude s'appuie sur les données de CSF‐2023, une enquête nationale transversale sur la sexualité en France. L'échantillon inclut 6305 individus sexuellement actifs âgés de 18 à 49 ans. Des régressions logistiques ont été menées pour identifier les facteurs influençant l'acceptabilité, la recherche d'informations et les comportements d'achat liés aux services SRH en ligne, dans divers domaines (contraception orale, avortement médicamenteux, traitement des IST/VIH et amélioration des performances sexuelles).

**Résultats:**

L'acceptation des hommes était plus élevée que les femmes (59% contre 53%), bien que l'acceptabilité de chaque service reste modeste (de 10% pour l'avortement médicamenteux à 23% pour la Prep). Les femmes recherchaient plus d'informations en ligne (75% contre 70%), en particulier sur la contraception et l'avortement, tandis que les hommes se concentraient davantage sur l'amélioration des performances sexuelles. Les personnes plus jeunes et plus éduquées, celles ayant plusieurs partenaires ou ayant rencontré des partenaires en ligne, cherchaient une gamme d'informations plus large. L'acceptabilité était aussi plus grande chez les hommes ayant des rapports avec des hommes et les répondants ayant subi des violences ou déclarant des grossesses non désirées, tandis que les résidents ruraux étaient moins acceptant et recherchaient moins d'information en ligne. Le recours aux soins médicaux récents diminuait l'acceptation, mais n'affectait pas la recherche d'informations.

**Conclusion:**

Cette étude montre un intérêt limité pour les services SRH numériques sans prescription en France, malgré une dépendance croissante aux plateformes numériques pour obtenir des informations. Des investissements ciblés sont nécessaires pour combler le fossé numérique, améliorer la qualité des services et explorer des sources alternatives pour répondre aux besoins d'autosoins en SSR.

## 1 Introduction

The World Health Organization (WHO) defines self‐care as “the ability of individuals, families, and communities to promote health, prevent disease, maintain health, and cope with illness and disability with or without the support of a healthcare provider.” [1, p. 13] Although self‐care has long been central to health management, its role in sexual and reproductive health (SRH) has only recently received scientific attention, in response to technological advancements [2] and growing momentum for patient autonomy in SRH care [3], contrasting with other areas of health, such as non‐communicable diseases (NCDs), where self‐care has been widely studied and promoted for decades.

Sexual and reproductive health self‐care lies at the intersection of daily life and health systems, building people's knowledge, skills, and resources to preserve their health (people‐centered) while promoting task‐sharing to extend the benefits of care delivered by SRH providers (system‐centered) [4]. The safety and efficacy of self‐care interventions are established across multiple SRH domains, including: (1) Knowledge generation on essential components of SRH through educational interventions (e.g., antenatal and postpartum care, contraception, sexually transmitted infections [STIs]); (2) Self‐management of prophylactic strategies (e.g., self‐use of contraceptive methods for pregnancy prevention, iron‐folic acid supplementation, Pre‐Exposure Prophylaxis [PreP] use for Human Immunodeficiency Virus [HIV], etc.); (3) Self‐testing (sexual transmitted infection [STI]/HIV, Human Papillomavirus [HPV, pregnancy]), self‐monitoring [ovulation kit, blood pressure or glucose during pregnancy]; and (4) Self‐treatment (STIs, medication abortion, and lubricants) [1, 5]. These self‐care practices fulfill an important role in promoting access and patient autonomy, yet their benefits need to be weighed against the risk of encouraging unregulated low‐quality, and non‐subsidized healthcare solutions [4]. This is particularly true in the contested field of SRH, where social norms constrain people's sexual identities, practices, and SRH service needs and legal restrictions limit access to affordable and confidential provider‐administered services [6]. As such, the WHO has developed a framework, situating self‐care practices within an ecosystem designed to enable the flow between SRH self‐management and provider‐administered care, acknowledging the expansion in places of care, including digital platforms, while stressing the importance of key principles to ensure equity and safety in self‐care strategies [7]. These principles follow a human rights approach emphasizing access, quality, informed choice, equality, privacy, and accountability [7].

Data on the adoption of certain SRH self‐care practices at the population level are well documented globally, such as coital‐dependent contraception and/or STI prevention methods (e.g., use of condoms, barrier methods, and emergency contraception). Evidence on the prevalence of other practices, such as self‐injectable contraception [8, 9, 10, 11] and self‐managed abortion [12, 13, 14] is emerging but remains largely confined to countries/regions where provider care is scarce or legally restricted, making self‐care a necessary substitute rather than an informed choice. Consequently, little research addresses high‐income contexts where SRH services are readily available, limiting our understanding of self‐care across the continuum of SRH care.

The rise of digital platforms has accelerated the uptake of SRH self‐care, as people increasingly turn to the Internet for SRH information, to monitor their health, and to seek SRH services [15]. Abundant online information contributes to SRH education and informs self‐management of symptoms, while digital apps help women and other pregnancy‐capable people track their periods and manage their fertility. Digital platforms also expand people's convenient and confidential access to self‐screening and self‐treatment options for STIs/HIV, contraception and abortion, sexual dysfunction, menopausal symptoms, and gender‐affirmative treatments. The digital SRH market has grown rapidly amid the COVID‐19 global pandemic, but the lack of access to technology, low digital literacy, and the lack of community infrastructure remain significant barriers, contributing to a global “digital divide” between and within countries that can amplify SRH inequities [16]. Existing research in high‐income settings consistently demonstrates disparities in online health information‐seeking behaviors across demographics, such as age, gender, and education, as well as by race/ethnicity and residence in the United States (US) [17, 18]. However, these studies generally neglect a specific focus on SRH, and most do not examine the connection between attitudes, information‐seeking, and self‐care practices outside of small‐scale mHealth interventions, which cannot be generalized to the general population [18]. Insights from the Global Values Survey, covering 112 countries (29% from the WHO European region), reveal substantial reliance on online information for reproductive health (61%) and sexual health (60%) due to its accessibility, convenience, and privacy. However, most participants preferred accessing SRH products from healthcare providers or pharmacies rather than online platforms. These findings underscore the importance of better understanding attitudes and behaviors related to digital SRH self‐care to grasp demand and practices, illuminate disparities, and discern whether online SRH resources supplement or replace traditional provider‐administered care.

In France, despite a legal tradition of embracing SRH self‐care solutions, authorizing over‐the‐counter access to emergency contraception in 1999, partial home abortions (use of misoprostol at home after receiving mifepristone from a healthcare provider) in 2004, access to PrEP in 2016, and allowing telemedicine medication abortion care in 2021, the country falls short on de‐medicalizing the full range of WHO‐recommended SRH self‐care solutions, including over‐the‐counter access to hormonal contraception, STI self‐treatment, and self‐management of both mifepristone and misoprostol for medication abortion. National surveys show a rise in general digital health information seeking between 2010 and 2014, followed by a decline in 2017, potentially reflecting decreased trust in online information [19]. Women, younger people, more educated, and wealthier individuals were the primary users [19]. These trends highlight digital health inequities, but their relevance to SRH is unclear, and pandemic‐related shifts in online health information‐seeking and self‐care behavior warrant further investigation.

This study sought to address some of these knowledge gaps surrounding SRH self‐care practices by: (1) Describing the social acceptability and use of digital resources for SRH among a large, representative sample of the population living in continental France; and (2) Identifying correlates of acceptability and usage of digital SRH resources according to one's gender, social position, residence, sexual experiences, and access to provider‐administered care. Together, the results provide insights into the social determinants of digital SRH usage to inform programs and policies designed to promote demand, access, and use of reliable and affordable digital SRH solutions as a complement to provider care.

## 2 Methods

### 2.1 Study Sample

The study draws on CSF‐2023 (Contexte des sexualités en France 2023), a national population‐based study on sexuality and sexual health in France, which seeks to explore trends in SRH health in the last three decades and explore contemporary issues in SRH, including the interplay between self and provider SRH care [20]. As detailed by Moreau and colleagues in this special issue [20], CSF‐2023 is a cross‐sectional study using random digit dialing to select a representative sample of the entire population aged 15–89 years residing in continental France. A total of 21,259 participants provided consent to participate in a phone interview (participation rate: 34%), among whom 12,906 participants aged 18–89 years completed an online survey extension (61% of the original sample), including information on self‐ and provider‐administered SRH care practices. We limited our analysis to sexually active adults of reproductive age (18–49 years) who had ever had sexual intercourse because individuals who were 50 years and older and individuals who never had sexual intercourse were ineligible for several SRH services (such as pregnancy prevention) and were less likely to have utilized these services in the past, given the relatively recent advent of digital health. From the 10,950 eligible people 18–49 who completed the phone survey, 6517 completed the online survey, and 6305 had no missing data on outcomes of interest. When comparing our analytic sample to the eligible sample of 10,950 adults 18–49 years who ever had sexual intercourse, we found similar distributions for all variables presented in Table 1 within margins of 0%–2%, except for pregnancy history and residency (3% difference) and religiosity (4% difference).

**TABLE 1:** Indicators of digital information seeking, attitudes, and practices for sexual and reproductive health (SRH) self‐care.

Domains	Initial items	Analyzed indicators (dichotomous)	Composite measure
Acceptability			
Do you favor access to the following SRH products online? Yes (with a prescription); Yes (without a prescription); No	Oral contraception	Contraception	Additive (0–4)
	Abortion pill	Abortion	
	Sexually transmitted infection (STI) treatment	STI treatment/Prep	
	Prep (HIV prophylaxis)		
	Treatment for Erection	Sexual performance	
Information‐seeking			
Have you ever sought information on the internet about the following topics?	Contraception	Contraception	Additive (0–4)
	Abortion	Abortion	
	STI	STI/HIV	
	HIV/AIDS		
	Problems related to sexuality	Sexuality	
Purchasing behavior			
Have you ever bought the following products on the internet?	Oral contraception	Contraception	
	Abortion pill	Abortion	
	Medication to improve erection	Sexual performance	
	Other products to enhance sexual performance		

We used initial survey weights to account for different probabilities of inclusion, and applied post‐stratification weights to the initial sample (sample of 21,259 respondents) to match the distribution of sample characteristics with census data, according to sex, age, education, number of household members, and region of residence. We further accounted for sample attrition (between participation in the phone and online surveys) by applying inverse probabilities of online survey participation, derived from a logistic regression model predicting the probability of online survey participation as a function of sociodemographic characteristics, sexual orientation, and number of partners in the last year.

### 2.2 Data Collection

Trained interviewers conducted phone interviews from November 2022 to October 2023 and solicited information about participants' sociodemographic characteristics, medical coverage, self‐rated health, and recent medical care visits. The phone questionnaire, which lasted 33 min (excluding the consent process), collected information on sexual attitudes, orientation, partners, and practices, as well as participants' pregnancy history, contraceptive behaviors, STIs, sexual violence, and sexual dysfunction. Subsequently, participants self‐completed an online survey within 4 weeks of the phone interview, facilitated through a personal link sent via email or text message. The online component, which lasted an average of 30 min, sought additional information about participants' attitudes and practices related to online SRH services. More detailed information about the CSF2023 survey and its methodology is published in Moreau and colleagues in this special issue [20] and can be found at www.csf.inserm.fr.

### 2.3 Ethics Statement

The CSF‐2023 protocol was approved by the Institut National de la Santé et de la Recherche Médicale (INSERM) ethical review board, the committee for the protection of human subjects from Marseille University, and the National Commission for Information Technology and Civil Liberties. All participants provided initial oral consent to participate in the phone survey and were sent written information about the study via SMS or email. After completing the phone survey, they provided oral consent to fill out the online questionnaire and confirmed their consent by checking the consent box before accessing the online survey.

### 2.4 Measures

#### 2.4.1 Dependent Variables

We examined three sets of outcomes: acceptability of online SRH services, online SRH information‐seeking, and online purchasing behavior. We pilot‐tested questions twice before finalization. For each outcome, we describe the initial items, domains, and the construction of analytical variables (Table 1).

- We assessed acceptability of digital SRH self‐care services across five domains: oral contraception, medication abortion, PrEP for HIV prevention, treatment for STIs, and treatment of erectile dysfunction. For each domain, respondents indicated whether online access should be available without a prescription, require a prescription, or not be available at all online. We used these responses to create two types of variables. First, we constructed a categorical variable with three levels: acceptable without prescription, acceptable with prescription, and not acceptable. Second, we developed a dichotomous variable contrasting respondents who favored non‐prescription access with all others, including those who required a prescription or considered online access unacceptable. This binary measure isolates support for self‐care models. To capture overall support for online SRH self‐care, we summed the number of domains where non‐prescription access was favored, resulting in a composite measure ranging from zero to four, with a mean of 1.04 (95% CI: 0.99–1.08).
- We measured online SRH information‐seeking by asking respondents whether they had searched online for information on contraception, abortion, STI/HIV, and sexuality issues. For each domain, we created binary indicators and then summed the number of domains where information was sought to construct a composite measure ranging from zero to four, with a mean of 1.84 (95% CI: 1.79–1.90).
- We assessed online purchasing behavior by asking respondents whether they had purchased prescription‐free SRH products online, including oral contraception, medication abortion pills, STI treatment or PrEP, and medications for erectile dysfunction. Due to the very low prevalence of online purchasing, we did not construct composite measures for this outcome.

#### 2.4.2 Independent Variables

The CSF‐2023 study collected rich information about sexuality and sexual health. In this study, we considered participants' sexual orientation, number of partners in the last 12 months, pregnancy history in the last 10 years (none/intended pregnancy[ies] only/at least one unintended), history of sexual violence (none/unwanted sexual contact/attempted or forced sex), and sexual dysfunction in the last year. We derived questions from previous French national surveys on sexuality to allow for trend analysis over the last 4 decades [21, 22]. We defined dysfunction impairing the respondent's sexuality in the last 12 months as any problems of libido, pain during sexual intercourse, lack of sexual pleasure, vaginal dryness for women or erectile dysfunction or premature ejaculation for men, considered by the respondents as a “problem for their sexuality” as stated in the response option. This indicator was used in previous national French sexuality surveys [23]. It is consistent with Diagnostic and Statistical Manual of Mental Disorders, Fifth Edition guidelines, which require not only the presence of symptoms, but also that these symptoms be associated with marked distress or interpersonal difficulty [24].

The final set of indicators pertained to the respondent's gender, age, and social position (i.e., education, financial situation, migration status, and race/ethnicity) to examine disparities in SRH self‐care practices. We measured gender by asking respondents if they were male, female, or something else, with further specification for those who did not identify as male or female. Only 2 respondents did not identify as male or female and were therefore not considered in this analysis. We also included the area of residence and whether respondents received any medical consultation in the last 12 months, which may reflect or condition access to provider care.

### 2.5 Analysis

We first used descriptive statistics (percentages, means) to examine the distribution of the study sample along demographic, sexuality, and provider care indicators. Second, we described the acceptability of access to online SRH services with or without a prescription, by health domain (contraception, abortion, STI/HIV treatment, and erectile dysfunction treatment). We also examined online information‐seeking behaviors and purchasing of products online by SRH domain. Third, we conducted univariate and multivariable logistic regression models to identify correlates of acceptability, information‐seeking, and purchasing behaviors for each health domain (contraception, abortion, STI/HIV treatment, and erectile dysfunction treatment). However, due to the small number of respondents who purchased abortion pills or STI products, we did not examine patterns of purchasing behaviors for these two SRH products. These results are detailed in the appendices due to the extensive data but are also summarized in a visual figure and within the text. In the last stage of the analysis, we used the additive measures of acceptability and information‐seeking to examine the number of SRH services deemed acceptable and the number of SRH topics for which respondents sought information online. We conducted a multivariable ordinal regression to evaluate gender differences in digital self‐care attitudes and information‐seeking across SRH domains, adjusting for other covariates. We stratified analyses by gender as we anticipated digital SRH self‐care acceptability, information‐seeking, and purchasing behaviors would differ by gender. We weighted all analyses (except for the ologit models, which do not allow survey weights in Stata) to account for unequal probability of inclusion, post‐stratification weights, and attrition. We conducted sensitivity analyses using linear regression models with survey weights to assess the robustness of our results. We performed analyses using Stata 17 software.

## 3 Results

We describe the sociodemographic characteristics of the sample in Table 2. Respondents were on average 34.5 years of age. They were equally divided across large cities, medium/small cities, and rural areas. Nine in ten respondents were born in France. Women were more likely than men to have attained higher education (48% vs. 42%) but reported more financial difficulty (46% vs. 42%). Most respondents (69% of men and 75% of women) had one sexual partner in the last year, and 9.8% of men and 13.0% of women had ever had same‐sex partners. Women were more likely than men to report any pregnancy in the last 10 years (43% vs. 40%). Women were also more likely than men to report ever experiencing sexual violence (47% vs. 16%) and suffered more from sexual dysfunction causing distress in the last 12 months (11% vs. 3%).

**TABLE 2:** Characteristics of CSF‐2023 18–49 years, sexually active who completed the online survey (*N* = 6305).

			Men		Women	
			*N*	%	*N*	%
Total			2807	47.1	3409	52.9
Sociodemographic indicators	Age	18–29	726	31.7	964	32.3
		30–39	957	34.6	1174	33.4
		40–49	1124	33.7	1359	34.3
	Education	< High school graduation	305	34.7	283	26.2
		High school graduation	523	23.9	635	25.8
		Some college education	889	26.3	1131	30.5
		Graduate education	1090	15.2	1446	17.4
	Perceived financial situation	Okay	1904	58.1	2210	53.8
		Difficult/very difficult	898	41.9	2210	46.2
	Place of birth	France	2618	90.7	3242	91.1
		Europe	53	1.5	97	2.4
		Africa	94	6.2	96	4.9
		America	19	0.8	37	1.2
		Asia	20	0.7	20	0.5
	Religiosity	Very/important	347	15.1	541	18.9
		Not important‐no religion	2454	84.9	2950	81.1
	Residence	Large city/suburb	1048	32.2	1249	30.9
		Medium or small size city	925	35.5	1204	38.1
		Rural	833	32.4	1042	30.9
Sexual and reproductive health indicators	Number of partners in the last 12 months	0	304	13.1	324	10.3
		1	2016	68.2	2652	74.9
		> 1	487	18.8	521	14.8
	Ever has same sex partnerships	No	2468	90.2	2988	87.0
		Yes	336	9.8	505	13.0
	Pregnancy history last 10 years	No pregnancy	1643	60.1	1980	56.7
		Intended pregnancies only	729	24.0	941	25.5
		Unintended pregnancy	365	15.9	537	17.9
	Sexual dysfunction causing distress	Yes	101	3.4	379	10.5
		No	2706	96.8	3118	90.2
	History of sexual violence	No	2387	83.7	1890	52.9
		Touching	130	4.9	404	11.7
		Attempted or forced sex	288	11.3	1196	35.4
Health care	Consulted a healthcare provider in the last 12 months	Yes	2188	74.9	2963	83.5
		No	619	25.1	534	16.5

We present the distributions of respondents' acceptability of SRH online services by prescription requirement, SRH information‐seeking, and online purchasing behaviors of prescription‐free SRH products in Figure 1.

**FIGURE 1:**
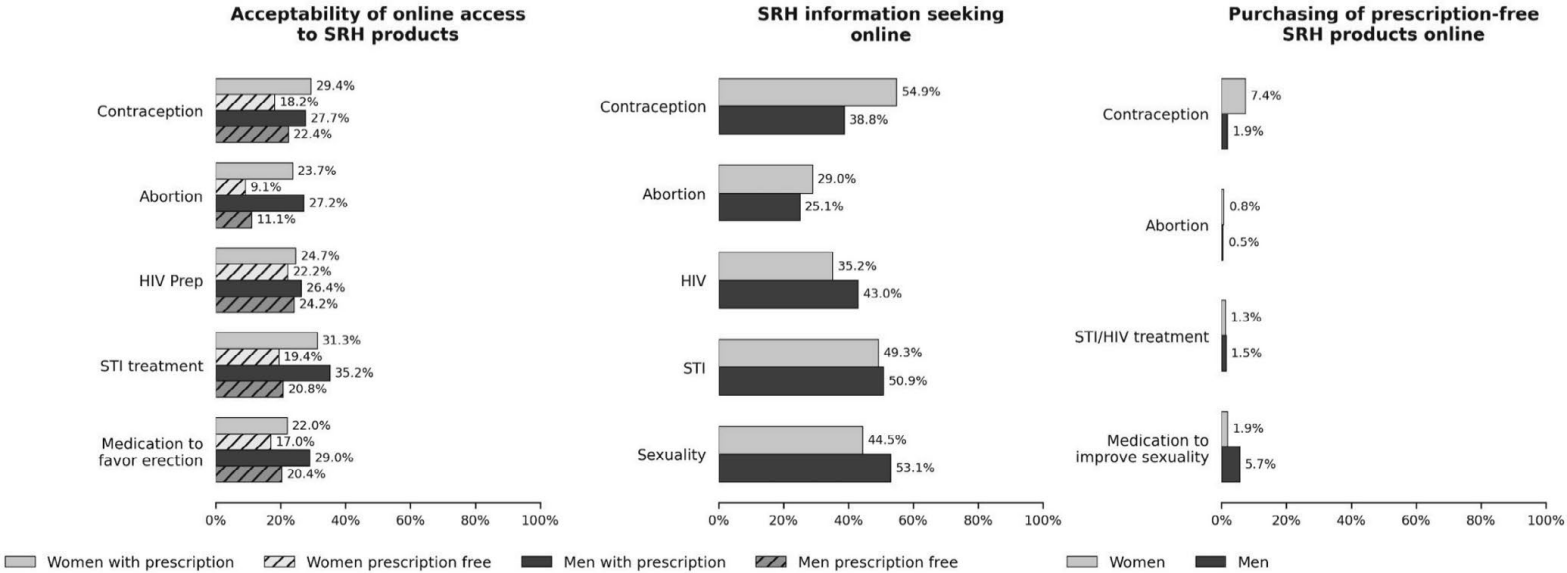
Percentage of self‐care acceptability, information‐seeking and purchasing by sexual and reproductive health domain and gender among CSF2023 respondents 18–49 years.

Acceptability of access to digital SRH products varied by gender, SRH domain, and prescription requirement (Figure 1). Men generally exhibited greater acceptance than women, and both genders preferred prescription over prescription‐free access, with the largest disparity pertaining to abortion pills and STI treatment for all respondents and to contraception for women. Across domains, acceptability of online prescription access ranged from 26% (HIV Prep) to 35% (STI treatment) among men and from 22% (treatment of erectile dysfunction) to 31% (STI treatment) among women, while support for prescription‐free access varied from 11% (medication abortion pills) to 22% (contraception) among men and from 9% (medication abortion pills) to 22% (HIV Prep) among women. Notably, close to one‐quarter of respondents supported prescription‐free access to HIV prophylaxis (24% of men and 22% of women), 22% of men and 18% of women supported prescription‐free access to oral contraception, and only 11% of men and 9% of women favored prescription‐free access to abortion pills. Approximately half of respondents had sought information online regarding sexuality, contraception (for women), or STIs, this proportion dropping to 39% for HIV and 27% for abortion (Figure 1). Women were more likely than men to seek digital information on contraception (55% vs. 39%, *p* < 0.001) and abortion (29% vs. 25%, *p* = 0.01), while men sought more information on sexuality (53% vs. 45%, *p* < 0.001). Online purchase of non‐prescription SRH products was low across indicators, rising to 7% for the contraceptive pill among female respondents.

### 3.1 SRH Self‐Care Correlates by Domains: Summary of Multivariable Logistic Regression Analysis

Table 3 summarizes all multivariable results across self‐care indicators (acceptability or information‐seeking) and SRH domains, while we provide more detailed findings in Appendices 1 and 2. Acceptability of SRH services varied by fewer sociodemographic characteristics compared to information‐seeking, which was more socially determined. Acceptability was unrelated to age and education, while information‐seeking increased with both. Rural residents were less accepting of contraception, abortion, and STI treatment, and rural men were less likely to seek online information about sexuality or STI treatment.

**TABLE 3:** Summary of factors associated with the acceptability of online prescription‐free sexual and reproductive health services across domains: Results for multivariable regression models among CSF‐2023 respondents 18–49 years.

		Contraception	Abortion	Sexually transmitted infection	Sexuality
Information seeking	Men	↓ Age ↑ Education ↓Born in Asia ↑ Unintended pregnancy ↑History of sexual violence	↓ Age, ↑Education ↑ Large urban ↑ Met partner online ↑ Unintended pregnancy ↑ History of sexual violence	↓ Age, ↑ Education ↑ Large urban ↑ Met partner online ↑ Intended pregnancy ↑ History of sexual violence	↓ Age, ↑ Education ↑ Large urban ↑ Met partner online ↑ Same sex partner ↑ Sexual dysfunction
	Women	↓ Age, ↑ Education ↑ Partnership12 months (m) ↓ Same sex partner ↓ Intended pregnancy ↑ History of sexual violence	↓ Age, ↑ Education ↑ Multipartnership 12 m ↑ Unintended pregnancy ↑ History of sexual violence	↓ Age, ↑ Education ↑ Multipartnership 12 m ↑ Met partner online ↓ Intended pregnancy ↑ History of sexual violence	↓ Age, ↑ Education ↑ Born in America ↑Partnerships 12 m ↑ Met partner online ↓ Same sex partner ↑Sexual dysfunction ↑ History of sexual violence
Acceptability	Men	↓ Rural ↓ 1 partner 12 m	↓ Rural ↓ Born in Europe	↓ Rural ↑ Born in Asia ↑ Same sex partner ↑ History of sexual violence ↓ Medical consultation 12 m	↓ Medical consultation 12 m
	Women	↓ Education ↑ History of sexual violence	↓ Age ↓ Rural	↓ Rural ↑ Multipartnerships 12 m	↑ Met partner online ↑ Sexual dysfunction ↓ Medical consultation 12 m

*Note:* ↓ signals statistically significant declines in the odds of online SRH information seeking or acceptability of prescription‐free access to online services. ↑ signals statistically significant increases in the odds of online SRH information seeking or acceptability of prescription‐free access to online services.

Self‐care acceptability showed little variation, while information‐seeking varied significantly by respondents' sexuality. Respondents who met their partners online and women who had more than one partner in the last year were more likely to seek information online across all SRH topics. Men who had ever had a same‐sex partner were more likely to seek online information about sexuality, while the reverse was true for women. Respondents who reported past sexual violence were more accepting of prescription‐free access to contraception (women) or STI treatment (men) online and more likely to seek information across a range of SRH topics. Likewise, respondents who experienced unintended pregnancies were more likely to seek information about contraception (women) and abortion.

Finally, women who had a recent medical consultation were less favorable to some online SRH services (i.e., STI treatment, sexuality), but regular care did not influence information seeking behaviors.

### 3.2 Cumulative Acceptability and Information Seeking Across SRH Domains

Looking across SRH domains, Figure 2 displays the distribution of the number of SRH services deemed acceptable and the number of SRH topics for which respondents sought information.

**FIGURE 2:**
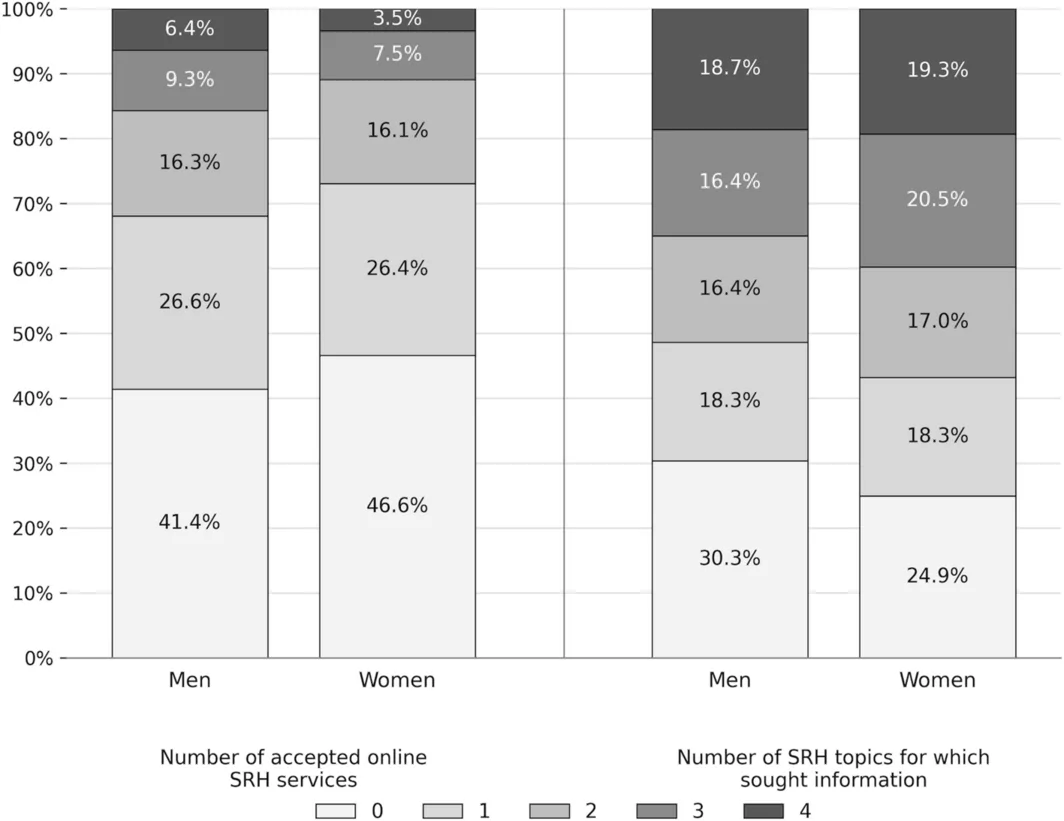
Number of online sexual and reproductive health services considered acceptable without prescription and number of topics researched for information among CSF2023 respondents 18–49 years.

Men were more accepting of at least one SRH online service than women (59% vs. 53%) and favored a higher average number of online SRH services (1.13 [1.06–1.20] vs. 0.95 [0.89–1.00], *p* = 0.03). Women were more likely to seek information about at least one topic compared to men (75% vs. 70%) and, on average, sought information on more topics than men (1.91 [1.84–1.98] vs. 1.75 [1.67–1.83], *p* = 0.04).

Multivariable ordinal regression models adjusting for all covariates (Table 4) confirmed women's lower acceptance of non‐prescription access to a diversity of SRH products online (proportional odds ratio (pOR) = 0.70 [0.63–0.77], *p* < 0.001) and greater information seeking across SRH topics (pOR = 1.19 [1.08–1.32], *p* < 0.001) compared to men, after adjustments. In addition, any additional positive attitude towards SRH online increased the proportional odds of seeking SRH information on an additional topic by 10% (pOR = 1.11 [1.06–1.15], *p* < 0.001). Stratified analysis by gender largely confirmed some of the patterns described in Table 3, while additional associations emerged. Younger respondents and highly educated respondents were more likely to seek information on a diversity of topics, as were those reporting multiple partners or meeting partners online. Men who reported same‐sex partnerships were also more accepting and sought more information on a diversity of topics. Likewise, respondents who reported sexual abuse were more accepting of and more likely to seek information on a diversity of SRH topics. Those who experienced unintended pregnancies also sought more information. Conversely, respondents from rural areas and those who reported religion was important in their lives were less accepting of and generally less likely to seek a diversity of SRH information online. Lastly, respondents who received recent medical care were less accepting of online SRH services, with no impact on information‐seeking. Sensitivity analysis using weighted linear regression models revealed similar patterns, although some associations were no longer significant, such as differences by region of birth and male same‐sex associations.

**TABLE 4:** Factors associated with levels of acceptability and information seeking across sexual and reproductive health topics among CSF2023 respondents 18–49 years: Results from multivariable ordinal logistic regression models.

		Acceptability				Information seeking			
		Men		Women		Men		Women	
		aOR	95% CI	aOR	95% CI	aOR	95% CI	aOR	95% CI
Age	18–29	Ref		Ref		Ref		Ref	
	30–39	0.99	0.81–1.21	0.89	0.74–1.06	**0.42**	**0.34–0.51**	**0.36**	**0.30–0.43**
	40–49	**1.21**	**1.0–1.46**	0.87	0.74–1.03	**0.21**	**0.17–1.25**	**0.19**	**0.16–0.23**
Education	< High school	Ref		Ref		Ref		Ref	
	High school	1.04	0.79–1.36	0.77	0.59–1.01	**1.41**	**1.08–1.84**	**1.87**	**1.43–2.43**
	Undergraduate	1.07	0.84–1.38	**0.70**	**0.55–0.90**	**1.78**	**1.39–2.28**	**2.57**	**2.01–3.29**
	Graduate	1.07	0.83–1.38	**0.71**	**0.55–0.90**	**2.23**	**1.74–2.87**	**3.42**	**2.67–4.39**
Difficult financial situation		1.13	0.96–1.32	1.05	0.91–1.20	1.13	0.97–1.32	1.00	0.87–1.24
Migration	Born French	Ref		Ref		Ref		Ref	
	Europe	1.00	0.67–1.51	1.28	0.87–1.90	0.74	0.49–1.10	1.08	0.73–1.60
	Africa	1.10	0.66–1.86	1.71	**1.20–2.45**	**0.81**	**0.49–1.34**	**1.61**	**1.09–2.35**
	America	**2.87**	**1.24–6.64**	1.81	0.98–3.35	1.11	0.47–2.61	1.53	0.81–2.87
	Asia	1.93	0.87–4.25	3.13	0.91–1.20	0.52	0.23–1.14	1.64	0.70–3.80
Religiosity	Important	**0.71**	**0.56–0.88**	0.88	0.73–1.06	**0.80**	**0.64–0.99**	**0.81**	**0.68–0.96**
Residence	Large city	Ref		Ref		Ref		Ref	
	Mid‐size city/town	0.90	0.76–1.06	0.96	0.83–1.12	**0.86**	**0.70_0.98**	0.94	0.81–1.09
	Rural area	**0.76**	**0.64–0.91**	**0.84**	**0.71–0.99**	**0.72**	**0.60_0.86**	0.86	0.73–1.01
Number of partners in last 12 months	0	Ref		Ref		Ref		Ref	
	1	1.03	0.82–1.32	1.13	0.90–1.42	1.07	0.84–1.36	**1.39**	**1.11–1.75**
	> 1	1.27	0.96–1.68	**1.33**	**1.01–1.76**	**1.54**	**1.17–2.03**	**2.10**	**1.61–2.75**
Met partners online		**1.20**	**1.04–1.40**	**1.28**	**1.12–1.47**	**1.48**	**1.28–1.71**	**1.50**	**1.31–1.71**
Same sex partners		**1.37**	**1.08–1.72**	1.17	0.97–1.41	**1.32**	**1.06–1.65**	0.98	0.82–1.18
Pregnancy history	Unintended	1.14	0.92–1.41	1.04	0.86–1.25	**1.83**	**1.47–2.28**	**1.60**	**1.33–1.93**
	Intended only	0.97	0.80–1.25	0.99	0.84–1.17	0.95	0.80_1.13	**0.83**	**0.71–0.97**
Sexual violence	Unwanted contact	1.33	0.99–1.77	**1.29**	**1.07–1.55**	1.27	0.95–1.70	**1.33**	**1.11–1.58**
	Assault	**1.31**	**1.02–1.68**	**1.29**	**1.11–1.50**	**1.40**	**1.09–1.81**	**1.63**	**1.41–1.89**
Sexual dysfunction last 12 months		1.13	0.78–1.61	1.02	0.83–1.24	1.19	0.83–1.70	**1.31**	**1.08–1.59**
Medical consultation last 12 months		**0.73**	**0.62–0.87**	**0.66**	**0.56–0.79**	1.17	0.99–1.38	0.97	0.81–1.15

*Note:* Proportional adjusted odds ratios and bolded values indicate statistical significance at *p* < 0.05.

## 4 Discussion

This study reveals nuanced patterns of digital SRH self‐care acceptability and practices in France. Online SRH information seeking was widespread (≈75%), whereas acceptability of digital SRH services was markedly low (≈75 unfavorable to any form of digital access to SRH services), and non‐prescription purchasing of SRH products online was rare (under 10%). Variations by gender, age, social background, sexual health experiences, and regular care patterns reveal a complex interplay of factors shaping digital SRH self‐care attitudes and practices. Taken together, results suggest limited opportunity for digital SRH self‐services to expand access and reduce inequity, but greater opportunities to strengthen SRH information provision, where engagement is already substantial.

Low acceptability of digital SRH self‐services appears rooted in general reluctance towards digital services and concerns about the safety of prescription‐free access. Favorable attitudes ranged from 35%–51% for prescription‐required services (e.g., medication abortion pills, STI treatments), but dropped to 10%–23% for prescription‐free access (abortion pills, PrEP). Although cross‐national comparisons are limited, our findings echo previous reports of a preference for in‐person SRH care over digital alternatives. For instance, the 2012 British National Survey of Sexual Attitudes and Lifestyles (Natsal) showed only 5% of men and 1% of women using contraceptives would have preferred obtaining their contraceptive supplies from a National Health Service or Department of Health website [25]. Similarly, a 2018 global values and preferences survey indicated a preference for provider care or pharmacy access over online access to SRH products [26]. Reasons for this reluctance are multifaceted, as highlighted in a qualitative study of young people's perspectives on online sexual health services [27]. They struggled with navigating numerous sources of information without clear recognition of trusted sources, and raised issues of confidentiality breaches [27]. With the expanded availability of digital health solutions post‐COVID‐19, it is critical to educate the public about trusted SRH digital services [28]. At the same time, alternative sources of self‐care, such as pharmacies (including mobile pharmacies in rural settings), should be promoted to disseminate recommended SRH self‐managed products [29].

In contrast, information‐seeking online was prevalent across SRH topics (e.g., 27% for abortion to 50% for STIs), although online purchasing of prescription products was rare (< 5%, rising to 7.4% for online oral contraception purchases for women). Again, comparisons are limited due to differences in indicators and time‐frames, but results from the Natsal study in Britain also report low rates of Internet use for seeking advice/help regarding sex lives, STI testing or treatment, and access to condoms/contraceptives over 12 months (7% for men and 5% for women) [25]. In the context of escalating legal barriers to abortion care in the United States, a national study conducted in 2017 estimated that 7% of women had ever self‐managed an abortion [30], which is significantly higher than our 0.7% estimate. However, the source of these abortions (online vs. other means) was not specified, limiting comparability. Notably, only 3% of women in our study who searched for abortion information online reported utilizing online services for self‐managed abortion, contrasting with the 28% reported in another US study of people searching for information about abortion online [31]. In France, factors such as cost and distance to care, which are common drivers of self‐managed abortion in the United States [12] before the 2022 *Dobbs v. Jackson Women's Health Organization* decision that eliminated federal protections for abortion rights, have less influence due to public insurance coverage for abortion and legal mandates to provide abortion care in all administrative regions. The enshrinement of abortion in the French constitution further affirms people's right to access provider‐administered abortion services, thereby limiting reliance on online unregulated services.

Digital information seeking was particularly common among respondents 18–29 years across all SRH topics, consistent with broader generational trends in digital health use [25, 32, 33]. This suggests opportunities to expand targeted online SRH education for young people, but also the need to confront existing obstacles related to the low acceptability of online SRH services, as suggested in our study, as well as the plethora of resources lacking clear indicators of quality [28] and privacy concerns [34]. Bridging this gap through engagement with provider‐administered care is essential to ensure legitimacy and establish connections with health systems for a seamless continuum of care [5, 35].

Gender differences in digital self‐care attitudes and practices were evident. Men generally displayed higher acceptability of online access to SRH services, while women engaged more in information‐seeking across a broader spectrum of SRH topics and utilized more prescription‐free online services. Attitudes and practices aligned with gender‐specific SRH needs; women showed greater acceptance, sought more information, and sought more services for oral contraception, whereas men exhibited higher acceptability, sought more information, and were more inclined to purchase sexuality‐enhancing medication. Gender patterns observed in the Natsal study in Britain were reversed, with men reporting greater use of the Internet for sexual health reasons than women, including higher usage of online contraceptive self‐care (men: 2%; women: 0.5%) [25]. However, it is worth noting differences in survey questions, wherein the Natsal survey encompassed contraceptive self‐care for any method, including condoms, whereas the French survey primarily focused on oral contraception.

Gender intersected with various social and sexual experiential factors among our nationally representative sample of participants, revealing some disparities in SRH self‐care practices. Like the Natsal study [25], urban living and higher education correlated with greater information‐seeking online. The educational gap likely reflects inequities in access and use of digital health technology highlighted in a recent WHO literature review across the European region [26]. Importantly, educational inequities are poised to amplify health disparities, particularly since our findings show digital information‐seeking does not compensate for the absence of recent access to provider care. Educational inequities intertwine with residential patterns, revealing lower acceptability and usage of digital SRH resources among rural residents. This occurs despite a rural–urban gradient in healthcare provider resources, which disadvantages rural residents in France [36]. Such disparities are highlighted in Wang and colleagues' 2021 meta‐analysis pointing to sociodemographic inequities in digital‐health seeking behaviors, alongside quality, trustworthiness, and utility of online health information [18]. Addressing these disparities is essential to harness the full potential of “digital care companionship” [28] by reducing barriers to internet access and promoting digital literacy while enhancing the quality of digital health content, through trusted channels and tailored information for different audiences. The promotion of reputable digital SRH sources is paramount to health education and to address the growing market of online counterfeit medicine, targeting “sexual enhancers” [37] or other medications (abortion pills, PrEP) [38]. These programmatic efforts should go hand‐in‐hand with policies expanding national health insurance coverage for digital SRH services to avoid shifting costs away from the health system to users [39].

Beyond demographic determinants of acceptability, information‐seeking, and purchasing practices, SRH self‐care was shaped by sexual orientation and experiences. Non‐heterosexual individuals displayed higher acceptance of online‐based SRH care, while people who met partners online or had more than one recent partner sought more information online across several SRH domains. Likewise, respondents who experienced adverse events, such as sexual violence or an unintended pregnancy in the last 10 years, also sought more information online. Prior studies have documented greater reliance on online SRH resources among men who have sex with men, and the Natsal study also suggested greater reliance on SRH digital health among individuals meeting partners online and those reporting condomless sex with new partners [25]. We extend these findings by showing that the internet is an important source of information for individuals experiencing adverse and stigmatized sexual and reproductive health events who may prioritize privacy and discretion in receiving SRH care. These digital platforms should offer the potential to bridge to in‐person SRH services to ensure that these individuals benefit from a continuum of care.

Our results should be interpreted with several limitations in mind. Questions on self‐care were limited given the multi‐thematic purpose of the CSF2023 study. Consequently, important topics, such as self‐care for menopause or gender affirming care, were not explored. Additionally, we did not factor in respondents' broader perspective on digital health, including their digital literacy or internet access, and failed to capture the reasons for declining or not using digital SRH services, including trustworthiness and perceptions of need for health services, which are major incentives for digital health‐seeking behaviors [18]. The selection of online survey participants partly addresses some of these barriers (e.g., literacy), but as such may limit the external validity of the study, although careful post‐stratification weighting was applied to account for sample selection. Limiting our sample to individuals 18–49 years who identified as male or female also limits the generalizability of our findings to younger or older populations as well as non‐binary individuals.

Because the random‐digit‐dialing design required minimizing early hang‐ups, especially given the broad age range of survey participants, we could not ask separate sex and gender items at the start of the interview, which has not been common practice in French surveys. Although later questions captured sexual identity and measures of masculinity/femininity, these constructs cannot substitute for self‐reported gender identity, including non‐binary gender. The sample size, while substantial, was still limited to examine differences by region of origin, and therefore our results should be considered as exploratory, warranting further exploration in minority populations who may have greater need but limited access to digital health solutions. Finally, the cross‐sectional nature of the survey limits the interpretation of the results, as the timing of self‐care practices did not coincide with the timing of attitudes or other correlates. In particular, the association between attitudes and information‐seeking is likely bi‐directional.

Despite these limitations, our study provides important insights into attitudes and practices related to SRH self‐care using digital platforms. The large population‐based sampling coupled with the specific focus on SRH services enhances the external and internal validity of the study, as reviews of digital health utilization indicate results are sensitive to population selection (mostly non‐population‐based) and topics of health inquiries [18]. Rich information on socio‐demographics and sexual lifestyles also offers a comprehensive understanding of the intersecting dimensions shaping digital SRH self‐care attitudes and behaviors.

Taken together, these findings indicate limited demand for digital SRH service delivery in France, but substantial engagement with digital SRH information. Policy efforts should therefore prioritize improving access to trusted, high‐quality SRH information, strengthening digital literacy, and developing clear pathways from online information to in‐person care through pharmacies, hotlines, or other community‐based channels. At the same time, research should focus on understanding the drivers of low acceptability of digital SRH services, evaluating hybrid models that combine digital information with provider‐linked care, and examining how digital SRH resources can be tailored to different demographic and social groups—particularly younger adults, those with lower education, rural residents, and individuals seeking privacy following stigmatized SRH experiences. Such work will be essential to ensure that digital SRH platforms contribute meaningfully to equitable SRH knowledge and access within the French healthcare context.

## Appendix 1 Factors Associated With Favorable Attitudes Towards Online Nonprescription Access to SRH Products According to Type of Products: Results From Multivariate Ordinal Logistic Regression Models

**Table:**

Attitudes			Contraception		Abortion		STI		Sexuality	
			Men	Women	Men	Women	Men	Women	Men	Women
Sociodemographics	Age	18–29	Ref	Ref	Ref	Ref	Ref	Ref	Ref	Ref
		30–39	0.84 (0.57–1.23)	0.99 (0.69–1.44)	0.90 (0.56–1.44)	0.71 (0.46–1.08)	0.95 (0.66–1.38)	0.96 (0.70–1.31)	1.11 (0.80–1.54)	0.93 (0.71–1.22)
		40–49	0.94 (0.66–1.36)	1.08 (0.77–1.52)	0.76 (0.48–1.22)	**0.63 (0.42–0.93)**	0.99 (0.71–1.39)	1.03 (0.78–1.38)	1.28 (0.95–1.73)	0.94 (0.73–1.20)
	Education	< High school graduation	Ref	Ref	Ref	Ref	Ref	Ref	Ref	Ref
		High school graduation	1.11 (0.73–1.67)	0.69 (0.47–1.02)	0.88 (0.53–1.50)	1.33 (0.73–2.41)	0.98 (0.67–1.42)	0.77 (054–1.10)	1.01 (0.71–1.43)	1.14 (0.82_1.60)
		Some college	1.04 (0.71–1.53)	**0.47 (0.32–0.68)**	0.90 (0.56–1.46)	1.27 (0.72–2.22)	0.89 (0.63–1.26)	0.73 (0.52–1.02)	1.06 (0.77–1.45)	1.07 (0.78–1.47)
		Graduate education	1.09 (0.73–1.62)	**0.54 (0.37–0.78)**	0.85 (0.52–1.40)	1.08 (0.61–1.92)	0.76 (0.52–1.10)	0.72 (0.51–1.01)	1.17 (0.83–1.63)	1.06 (0.76–1.47)
	Perceived financial situation	OK	Ref	Ref	Ref	Ref	Ref	Ref	Ref	Ref
		Difficult/very difficult	0.98 (0.73–1.31)	1.28 (0.98–1.67)	1.27 (0.87–1.86)	0.99 (0.71–1.38)	1.12 (0.85–1.47)	1.01 (0.80–1.26)	1.16 (0.90–1.50)	0.92 (0.74–1.13)
	Migration	Born French	Ref	Ref	Ref	Ref	Ref	Ref	Ref	Ref
		Europe	0.59 (0.27–1.28)	0.67 (0.31–1.46)	**0.22 (0.06–0.79)**	0.80 (0.28–2.29)	0.60 (0.30–1.21)	1.25 (0.67–2.32)	1.04 (0.57–1.89)	0.96 (0.86–2.10)
		Africa	1.21 (0.50–2.90)	1.72 (0.88–3.37)	1.06 (0.26–4.36)	0.80 (0.30–2.11)	0.87 (0.38–2.00)	1.35 (0.74–2.47)	1.62 (0.72–3.64)	1.41 (0.80–2.48)
		America	3.05 (0.83–11.21)	1.48 (0.61–3.59)	0.95 (0.21–4.27)	1.08 (0.37–3.14)	1.52 (0.37–6.30)	1.68 (0.69–4.07)	1.45 (0.32–6.66)	1.24 (0.47–3.25)
		Asia	0.56 (0.16–1.96)	4.37 (1.38–13.80)	1.07 (0.28–4.11)	0.74 (0.15–3.57)	**4.72 (1.57–14.15)**	2.88 (0.79–10.40)	2.06 (0.70–6.04)	1.15 (0.34–3.85)
	Religiosity	Religion not important	Ref	Ref	Ref	Ref	Ref	Ref	Ref	Ref
		Religion important	0.95 (0.60–1.50)	1.34 (0.94–1.93)	1.17 (0.67–2.04)	0.70 (0.41–1.22)	1.05 (0.70–1.56)	0.97 (0.70–1.33)	071 (0.49–1.01)	0.87 (0.74–1.12)
	Residence	Large city/suburb	Ref	Ref	Ref	Ref	Ref	Ref	Ref	Ref
		Medium or small size city	1.03 (0.74–1.43)	0.99 (0.71–1.38)	0.69 (0.44–1.07)	0.84 (0.58–1.22)	0.85 (0.63–1.15)	0.79 (0.61–1.03)	0.95 (0.72–1.25)	0.93 (0.73–1.18)
		Rural	**0.66 (0.46–0.95)**	1.01 (0.72–1.43)	**0.50 (0.31–0.81)**	**0.60 (0.40–0.89)**	**0.61 (0.43–0.86)**	**0.69 (0.52–0.91)**	0.83 (0.61–1.13)	0.98 (0.76–1.26)
SRH indicators	No of partners 12 months	0	Ref	Ref	Ref	Ref	Ref	Ref	Ref	Ref
		1	**0.55 (0.35–0.86)**	1.15 (0.68–1.92)	0.82 (0.43–1.55)	1.18 (0.69–2.04)	1.11 (0.72–1.72)	1.27 (0.83–1.94)	0.82 (0.56–1.19)	1.25 (0.863–1.81)
		> 1	0.79 (0.48–1.30)	1.01 (0.72–1.43)	1.03 (0.54–2.00)	1.22 (0.67–2.24)	1.38 (0.86–2.22)	**1.72 (1.05–2.81)**	1.21 (0.78–1.88)	1.38 (0.89–2.14)
	Met partner online	Yes	1.09 (0.81–1.49)	1.32 (0.99–1.74)	1.08 (0.72–1.63)	0.91 (0.64–1.28)	0.93 (0.71–1.22)	1.03 (0.82–1.31)	0.95 (0.75–1.20)	**1.30 (1.05–1.59)**
		No	Ref	Ref	Ref	Ref	Ref	Ref	Ref	Ref
	Same sex partnerships	No	Ref	Ref	Ref	Ref	Ref	Ref	Ref	Ref
		Yes	1.27 (0.84–1.90)	1.05 (0.73–1.51)	1.50 (0.90–2.51)	1.29 (0.89–1.87)	**1.52 (1.05–2.19)**	1.21 (0.89–1.65)	1.10 (0.76–1.59)	1.28 (095–1.71)
	Pregnancy last 10 years	No pregnancy	Ref	Ref	Ref	Ref	Ref	Ref	Ref	Ref
		Unintended pregnancy	1.14 (0.75–1.75)	1.10 (0.76–1.58)	0.80 (0.46–1.40)	0.70 (0.45–1.08)	1.37 (0.93–2.04)	1.07 (0.79–1.45)	1.09 (0.76–1.56)	1.02 (0.77–1.34)
		Intended pregnancy only	1.31 (0.55–1.05)	1.17 (0.81–1.67)	1.33 (0.83–2.14)	0.91 (0.58–1.41)	1.10 (0.80–1.52)	0.98 (0.72–1.32)	0.96 (0.72–1.28)	1.12 (0.86–1.44)
	Sexual dysfunction causing distress	Yes	0.80 (0.42–1.53)	0.86 (0.58–1?28)	0.56 (0.27–1.17)	0.79 (0.45–1.40)	0.68 (0.38–1.24)	1.07 (0.77–1.48)	1.28 (0.71–2.29)	**1.43 (1.06–1.92)**
		No	Ref	Ref	Ref	Ref	Ref	Ref	Ref	Ref
	History of sexual violence	No	Ref	Ref	Ref	Ref	Ref	Ref	Ref	Ref
		Touching	0.77 (0.44–1.32)	1.33 (0.93–1.92)	0.91 (0.44–1.88)	1.13 (0.74–1.74)	1.38 (0.80–2.38)	1.33 (0.98–1.80)	1.38 (0.79–2.39)	1.26 (0.95–1.66)
		Attempted or forced sex	1.14 (0.93–1.85)	**1.39 (1.03–1.88)**	1.16 (0.67–2.00)	1.24 (0.86–1.78)	**1.56 (1.03–2.35)**	1.19 (0.92–1.53)	1.47 (0.98–2.21)	1.12 (0.86–1.44)
	Consulted a healthcare provider in the last 12 months	Yes	0.76 (0.55–1.05)	0.83 (0.58–1.18)	0.82 (0.53–1.26)	0.69 (0.45–1.06)	**0.70 (0.52–0.96)**	0.70 (0.52–0.93)	**0.62 (0.47–0.82)**	**0.63 (048–0.82)**
		No	Ref	Ref	Ref	Ref	Ref	Ref	Ref	Ref

*Note:* Proportional adjusted odds ratios and bolded values indicate statistical significance at *p* < 0.05.

## Appendix 2 Factors Associated With Online Information Seeking by SRH Topic: Results From Multivariate Ordinal Logistic Regression Models

**Table:**

Information seeking			Contraception		Abortion		STI		Sexuality	
			Men	Women	Men	Women	Men	Women	Men	Women
Sociodemographics	Age	18–29	Ref	Ref	Ref	Ref	Ref	Ref	Ref	Ref
		30–39	**0.39 (0.27–0.54)**	**0.42 (0.31–0.58)**	**0.43 (0.30–0.61)**	**0.51 (0.38–0.69)**	**0.67 (0.47–0.95)**	**0.64 (0.47–0.87)**	**0.51 (0.36–0.75)**	**0.47 (0.35–0.63)**
		40–49	**0.18 (0.13–0.25)**	**0.17 (0.13–0.22)**	**0.20 (0.14–0.29)**	**0.23 (0.17–0.31)**	**0.36 (0.26–0.51)**	**0.42 (0.32–0.55)**	**0.37 (0.26–0.51)**	**0.28 (0.21–0.36)**
	Education	< High school graduation	Ref	Ref	Ref	Ref	Ref	Ref	Ref	Ref
		High school graduation	**1.87 (1.26–2.76)**	**2.10 (1.45–3.04)**	**1.72 (1.09–2.69)**	**1.88 (1.18–3.00)**	1.27 (0.88–1.84)	**1.85 (1.32–2.61)**	1.21 (0.84–1.74)	**1.48 (1.03–2.12)**
		Some college	**2.44 (1.70–3.50)**	**2.83 (2.00–3.04)**	**2.14 (1.42–3.24)**	**2.26 (1.44–3.56)**	**1.54 (1.11–2.15)**	**2.09 (1.51–2.88)**	**1.57 (1.13–2.19)**	**2.12 (1.51–2.97)**
		Graduate education	**3.42 (2.360–4.96)**	**5.52 (53.15–6.49)**	**2.24 (1.46–3.43)**	**2.51 (1.59–3.94)**	**1.86 (1.33–2.1§2)**	**2.82 (2.03–3.93)**	**1.98 (1.40–2.80)**	**2.77 (1.95–3.92)**
	Perceived financial situation	OK	Ref	Ref	Ref	Ref	Ref	Ref	Ref	Ref
		Difficult/very difficult	1.13 (0.86–1.48)	0.84 (0.68–1.06)	1.20 (0.90–1.61)	0.90 (0.71–1.14)	1.17 (0.89–1.53)	0.93 (0.75–1.16)	1.17 (0.89–1.53)	1.05 (0.85–1.31)
	Birth place	Born French	Ref	Ref	Ref	Ref	Ref	Ref	Ref	Ref
		Europe	0.77 (0.37–1.60)	0.97 (0.48–1.96)	1.64 (0.81–3.31)	0.91 (0.48–1.74)	1.01 (0.56–1.84)	1.13 (0.56–2.29)	0.94 (0.52–1.69)	1.63 (0.78–3.39)
		Africa	1.01 (0.30–3.37)	1.10 (0.58–2.09)	1.00 (0.41–2.40)	1.08 (0.57–2.07)	0.76 (0.38–1.56)	1.83 (0.87–3.85)	1.45 (0.54–3.91)	1.47 (0.85–2.56)
		America	1.11 (0.33–3.79)	0.99 (0.42–2.33)	0.68 (0.20–2.32)	1.11 (0.48–2.57)	2.64 (0.61–11.51)	1.31 (0.49–3.51)	0.62 (0.16–2.44)	**2.91 (1.00–8.48)**
		Asia	**0.28 (0.09–0.91)**	0.91 (0.34–2.44)	0.93 (0.28–3.07)	1.27 (0.36–4.55)	0.40 (0.14–1.12)	1.30 (0.45–3.77)	0.42 (0.16–1.09)	1.50 (0.58–3.90)
	Religiosity	Religion not important	Ref	Ref	Ref	Ref	Ref	Ref	Ref	Ref
		Religion important	0.78 (0.51–1.21)	1.13 (0.82–1.57)	0.71 (0.441–1.14)	0.91 (0.71–1.14)	0.98 (0.67–1.42)	1.05 (0.78–1.42)	0.79 (0.54–1.15)	1.01 (0.75–1.36)
	Residence	Large city/suburb	Ref	Ref	Ref	Ref	Ref	Ref	Ref	Ref
		Medium or small size city	0.82 (0.61–1.10)	0.81 (0.62–1.06)	**0.72 (0.52–0.99)**	0.87 (0.67–1.13)	**0.55 (0.41–0.75)**	0.90 (0.70–1.163)	**0.70 (0.52–0.94)**	0.96 (0.75–1.24)
		Rural	0.75 (0.54–1.04)	0.98 (0.74–1.31)	0.80 (0.56–1.4)	0.79 (0.59–1.07)	**0.54 (0.39–0.74)**	0.94 (0.72–1.22)	**0.68 (0.49–0.94)**	1.00 (0.77–1.30)
SRH indicators	No of partners 12 months	0	Ref	Ref	Ref	Ref	Ref	Ref	Ref	Ref

1		0.83 (0.56–1.23)	**1.49 (1.01–2.21)**	0.96 (0.62–1.48)	1.20 (0.75–1.92)	0.76 (0.51–1.13)	1.21 (0.81–1.81)	0.83 (0.56–1.23)	**1.56 (1.07–2.29)**

> 1	1.00 (0.63–1.62)	**2.38 (1.42–4.00)**	0.98 (0.58–1.64)	**1.83 (106–3.17)**	1.32 (0.81–2.17)	**2.25 (1.36–3.73)**	1.22 (0.76–1.96)	**2.98 (1.87–4.75)**

Met partner online	Yes	1.11 (0.87–1.43)	**1.48 (1.18–1.87)**	**1.46 (1.11–1.91)**	1.25 (0.97–1.61)	**1.50 (1.17–1.92)**	**1.31 (1.05–1.64)**	**1.31 (1.02–1.68)**	**1.52 (1.22–1.89)**

No	Ref	Ref	Ref	Ref	Ref	Ref	Ref	Ref

Same sex partnerships	No	Ref	Ref	Ref	Ref	Ref	Ref	Ref	Ref

Yes	0.75 (0.51–1.11)	**0.65 (0.46–0.91)**	0.85 (0.55–1.30)	0.99 (0.73–1.36)	1.37 (0.91–2.08)	1.25 (0.90–1.74)	**2.53 (1.60–3.98)**	**0.82 (0.60–1.13)**

Pregnancy last 10 years	No pregnancy	Ref	Ref	Ref	Ref	Ref	Ref	Ref	Ref

	Unintended pregnancy	**1.75 (1.18–2.60)**	1.31 (0.95–1.79)	**2.79 (1.88–4.13)**	**3.87 (2.80–5.35)**	1.22 (0.85–1.76)	0.88 (0.64–1.21)	1.11 (0.75–1.64)	0.86 (0.63–1.16)

Intended pregnancy only	0.97 (0.71–1.33)	**0.73 (0.57–0.96)**	**0.70 (0.49–1.00)**	**0.59 (0.43–0.80)**	**0.70 (0.53–0.94)**	**0.57 (0.44–0.75)**	1.01 (0.75–1.36)	0.82 (0.63–1.07)

Sexual dysfunction causing distress	Yes	0.80 (0.46–1.39)	1.16 (0.82–1.64)	0.99 (0.51_1.93)	1.07 (0.75–1.51)	0.82 (0.46–1.43)	1.22 (0.88–1.69)	**2.39 (1.15–4.94)**	**1.88 (1.33–2.66)**

No	Ref	Ref	Ref	Ref	Ref	Ref	Ref	Ref

History of sexual violence	No	Ref	Ref	Ref	Ref	Ref	Ref	Ref	Ref

	Unwanted contact	1.03 (0.60–1.80)	1.13 (0.84–1.52)	**2.03 (1.09–3.79) (1.02–3.74)**	1.10 (0.81–1.49)	1.63 (0.93–2.86)	1.00 (0.75–1.32)	1.13 (0.64–1.97)	**1.36 (1.03–1.80)**

Attempted or forced sex	**1.84 (1.10–3.12)**	**1.77 (1.34–2.30)**	**2.09 (1.29–3.40)**	**1.80 (1.37–2.38)**	**2.06 (1.28–3.31)**	**1.46 (1.13–1.88)**	1.82 (0.75–1.34)	**1.95 (1.53–2.51)**

Consulteda healthcare provider in the last 12 months	Yes	1.28 (0.95–1.73)	0.90 (0.67–1.21)	0.96 (0.69–1.33)	1.15 (0.82–1.61)	1.27 (0.95–1.69)	1.02 (0.77–1.35)	1.33 (0.99–1.79)	0.94 (0.70–1.25)

No	Ref	Ref	Ref	Ref	Ref	Ref	Ref	Ref

*Note:* Proportional adjusted odds ratios and bolded values indicate statistical significance at *p* < 0.05.
